# A patient-specific echogenic soft robotic left ventricle embedded into a closed-loop cardiovascular simulator for advanced device testing

**DOI:** 10.1063/5.0203653

**Published:** 2024-05-28

**Authors:** Maria Rocchi, Konstantina Papangelopoulou, Marcus Ingram, Youri Bekhuis, Guido Claessen, Piet Claus, Jan D'hooge, Dirk W. Donker, Bart Meyns, Libera Fresiello

**Affiliations:** 1 Department of Cardiovascular Sciences, KU Leuven, Leuven, Belgium; 2 Department of Cardiovascular Diseases, University Hospitals Leuven, Belgium; 3 Faculty of Medicine and Life Sciences, LCRC, UHasselt, Biomedical Research Institute, Diepenbeek, Belgium; 4 Hartcentrum Hasselt, Jessa Ziekenhuis, Belgium; 5 Cardiovascular and Respiratory Physiology, University of Twente, Enschede, The Netherlands; 6 Intensive Care Center, University Medical Center Utrecht, Utrecht, the Netherlands; 7 Department of Cardiac Surgery, University Hospitals Leuven, Leuven, Belgium

## Abstract

Cardiovascular medical devices undergo a large number of pre- and post-market tests before their approval for clinical practice use. Sophisticated cardiovascular simulators can significantly expedite the evaluation process by providing a safe and controlled environment and representing clinically relevant case scenarios. The complex nature of the cardiovascular system affected by severe pathologies and the inherently intricate patient–device interaction creates a need for high-fidelity test benches able to reproduce intra- and inter-patient variability of disease states. Therefore, we propose an innovative cardiovascular simulator that combines *in silico* and *in vitro* modeling techniques with a soft robotic left ventricle. The simulator leverages patient-specific and echogenic soft robotic phantoms used to recreate the intracardiac pressure and volume waveforms, combined with an *in silico* lumped parameter model of the remaining cardiovascular system. Three different patient-specific profiles were recreated, to assess the capability of the simulator to represent a variety of working conditions and mechanical properties of the left ventricle. The simulator is shown to provide a realistic physiological and anatomical representation thanks to the use of soft robotics combined with *in silico* modeling. This tool proves valuable for optimizing and validating medical devices and delineating specific indications and boundary conditions.

## INTRODUCTION

Cardiovascular medical devices are subject to extensive testing and analysis before their introduction into clinical practice. The preclinical testing phase has the primary goal of assessing the safety, efficacy, and potential risks associated with the medical device.^1^ Tests done during this phase include usability testing, biocompatibility testing, sterility testing, shelf-life and aging studies, and performance testing.^2^ The specific development and approval process can vary significantly depending on the complexity of the device, its classification, and regulatory requirements defined by agencies like the Food and Drug Administration (FDA) or the European notified bodies. Notably, in recent years, both the FDA and the European-notified bodies have strongly recognized the value of *in vitro* and *in silico* testing in the evaluation of medical devices,^3^ also reflecting their commitment to the principle of replacement, reduction, and refinement (3Rs) of *in vivo* testing.^4^

Simulation provides a safe and controlled environment to represent clinically relevant case scenarios allowing for feedback and assessment without putting real patients at risk. Physical and computer simulators can expedite the evaluation process, by improving and optimizing the medical device design iteratively, exploring complex patient–device interactions, and providing a deeper understanding of complex physiological processes and disease mechanisms before the actual testing on experimental animals or humans.^5^

A wide variety of cardiovascular simulators can be found in the literature, ranging from *in silico* simulators to numerically evaluate medical devices to physical mock loops to provide a tangible and controlled environment for experimental assessments. *In silico* systems refer to computational models that have the capability of simulating the cardiovascular system numerically.^6–10^ They proved to be valuable tools to model and simulate complex physiological processes, disease mechanisms, and the interactions of medical devices with the cardiovascular system.^6,7^ Thanks to their high flexibility and tunability, *in silico* systems contribute to time and cost efficiency, allowing rapid testing and evaluation of diverse scenarios, and ultimately facilitating iterative design and optimization of the medical device. Additionally, *in silico* simulations support customization based on patient-specific data, aiding in the development of tailored treatments. Nevertheless, this category of cardiovascular simulators finds its limitations when evaluating a physical medical device. In fact, the operation of the medical device has to be modeled, leading to a simplification or a neglect of some complex aspects, such as mass and momentum, of the device in the dynamic cardiovascular system.

*In vitro* models overcome the limitation of *in silico* models, as they are fully physical simulators connected to the medical device. They proved to be a meaningful translational tool to explore medical devices, and their complexity depends on the application.^11^ The first generation of mock loops recreated the hemodynamics of the cardiovascular site of interest in stiff hydraulic chambers, neglecting its geometry. Such mock loops have been used to test, e.g., mechanical circulatory support systems^12–14^ and heart valves,^15–17^ from a hemodynamic point of view. With the advance in 3D printing technologies and soft robotics, *in vitro* simulators have started including a more realistic anatomical geometry and/or mechanical properties of the pertinent cardiovascular site, broadening the testing of medical devices also to imaging techniques.^11^ Examples of such mock loops available in the market are the physiologic left ventricle test system of DesignPlex Biomedical LLC (Forth Worth, Texas, USA)^18^ and the pulse duplicator system of ViVitro Labs (Victoria, BC).^19^ Those simulators are developed to test not only specific medical devices, such as percutaneous mechanical circulatory support systems and heart valve prosthesis, but also ultrasound and magnetic resonance imaging techniques.^20–25^ These systems reproduce physiological waveforms of the pressure and/or volume in a silicon model of the left ventricle, but without including a representation of the remaining cardiovascular system. As such, these simulators can only evaluate the impact of the medical device locally on the left ventricle, without accounting for the surrounding cardiovascular system.

Consequently, other research groups have developed more sophisticated physical simulators that combine the compliant anatomical phantoms with a mock loop of the closed-loop circulation.^25–27^ However, these mock loops rely on the compliance of the material used for the anatomical phantom, whose value is not representative of the dynamically evolving myocardial stiffness during the relaxation and contraction phases.^11^ Some simulators employ soft robotics solutions to mimic the mechanical properties of anatomical sites. Singh *et al.*^28^ recreated the ventricular contraction by replacing the myocardium of an *ex vivo* pig heart with McKibben-style soft robotic actuators. While this setup achieves a good level of realism, the use of an animal heart restricts the simulation to non-patient-specific scenarios. Rosalia *et al.*^27^ used a silicon left ventricle with a soft robotic sleeve to replicate systolic contraction by compressing the element externally. Such a method could reproduce waveforms in agreement with *in vivo* experiments. However, the systolic contraction is not sufficiently realistic limiting the testing application of such a simulator. In general, these simulators require the implementation of many hardware elements, thus lacking the flexibility that is essential to guarantee the representation of different pathophysiological conditions.

Given the need in addressing the intra- and inter-patient variability for medical device testing, this study presents a new cardiovascular test bench that combines an *in silico* representation of patient-specific (patho-)physiological hemodynamics with a realistic left ventricular soft robot, for a concomitant high-fidelity anatomical and physiological simulation. The simulator is a combination of *in vitro* and *in silico* modeling techniques. It leverages the flexibility and tunability of *in silico* models, and it includes realistic *in vitro* anatomical elements to connect medical devices. The cardiovascular simulator includes a closed-loop cardiovascular system and an echogenic physiologically activated soft robotic 3D left ventricle able to reproduce realistic ventricular pressure and volume profiles.

## RESULTS

### Personalization of the hybrid simulator

**FIG. 1.**
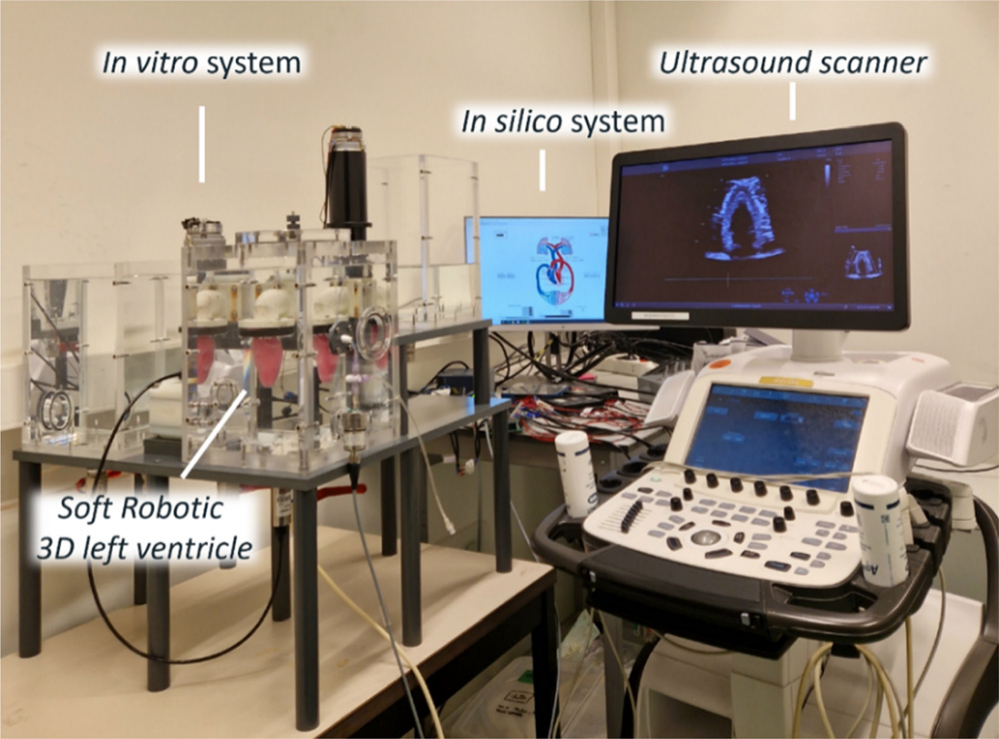
Hybrid cardiovascular simulator. The simulator combines an *in silico* representation of the closed-loop cardiovascular system with an *in vitro* system implementing a soft robotic 3D left ventricle. In the *in vitro* system the pressure and volume of the left ventricle are reproduced with high fidelity. The *in vitro* and the *in silico* systems exchange pressure, flow, and volume data in real-time under LabVIEW environment. The soft robotic 3D left ventricle is ultrasound (US) compatible, thus allowing assessment with an US scanner.

**FIG. 2.**
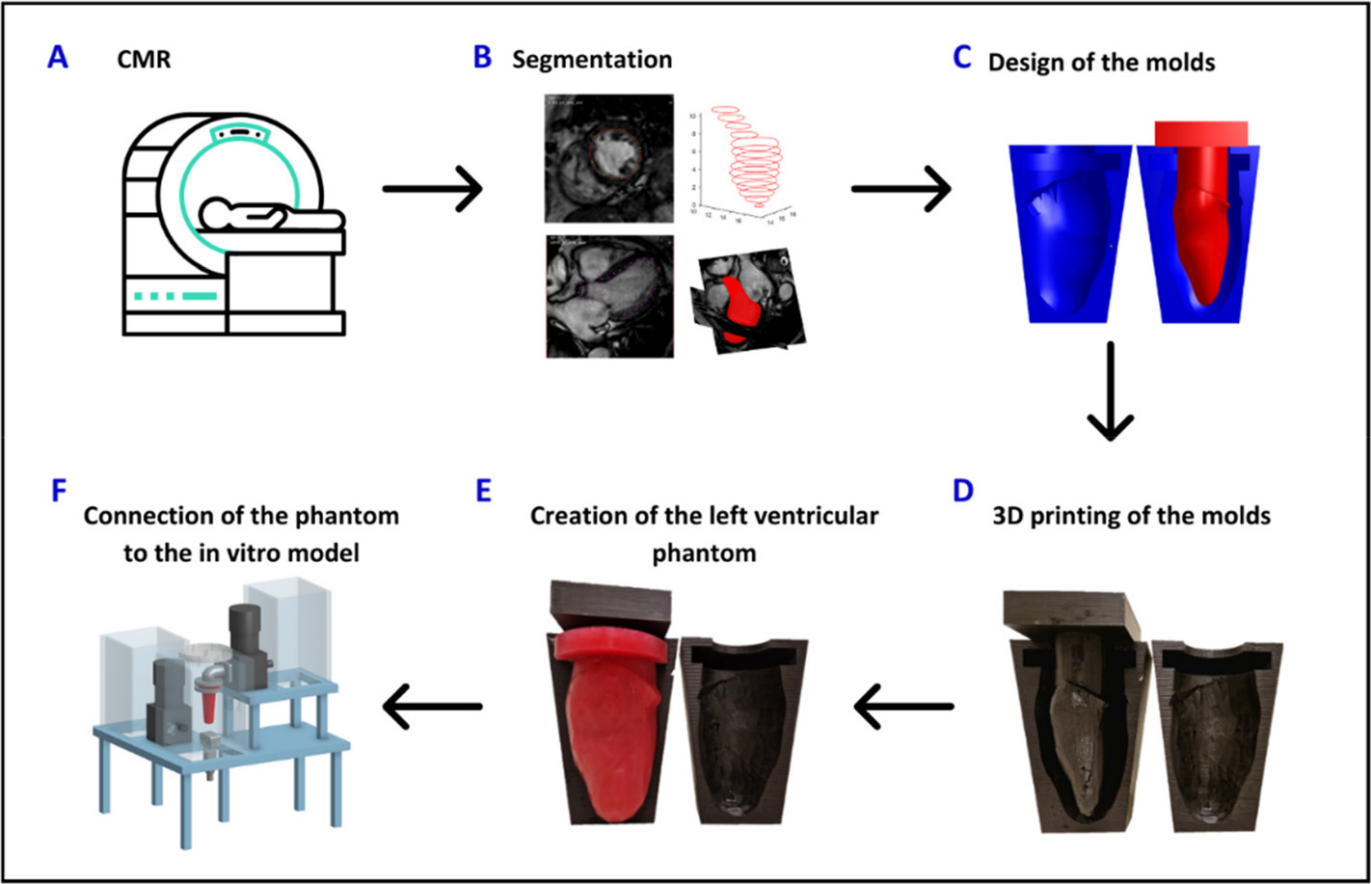
Workflow for the creation of the soft robotic left ventricle. Starting from the cardiac magnetic resonance (CMR) images of a patient (a), the anatomy of the left ventricle is extracted with a process of segmentation (b) so to design the molds (c). The molds are then 3D printed (d) and used to create the left ventricular phantom using the mold casting technique (e). The so obtained left ventricular phantom is then connected to the in vitro model (f).

We developed a hybrid simulator combining an *in silico* physiological model of the cardiovascular system with an *in vitro* soft robotic patient-specific left ventricle, as shown in Fig. 1. The two systems exchange pressure, flow, and volume data in real-time under LabVIEW environment (LabVIEW 2019 SP1, National Instruments, Austin, Texas). The hybrid simulator was tuned on the hemodynamic profiles of three patients,' retrospectively selected with the purpose of accounting for different (patho-)physiologies and left ventricular mechanical properties, in order to cover a diverse set of functional and structural characteristics. Following the workflow shown in Fig. 2, the left ventricular phantoms were realized in polyvinyl alcohol (PVA) using a mold casting technique. Finally, the material properties of each ventricle were characterized by imposing controlled volume changes in the phantom and acquiring the resulting transmural pressure values.

The compliance for each soft robotic left ventricle was calculated as a second-order regression line between the internal phantom volume and the transmural pressure as shown in Fig. 6 in the Appendix. Differences in the curves can be linked to the geometry of the left ventricular phantoms and/or the number of freeze/thaw cycles to which they were subject.

The *in silico* system consists of a closed loop lumped parameter representation of atria, right ventricle, heart valves, and pulmonary and systemic circulations. For each patient profile, the *in silico* model was personalized, in terms of heart rate (HR), mechanical properties of the right ventricle, and systemic and pulmonary circulatory parameters to match the hemodynamics of the patient.

### Simulated patients' profiles and comparison to clinical data

The *in silico* cardiovascular model computes the hemodynamics of each cardiovascular chamber (left and right atrium, right ventricle, and systemic and pulmonary circulations) in real-time with a temporal resolution of 1 ms. Only the left ventricle is reproduced *in vitro* through a physiologically activated soft robotic phantom. This is done by considering the preload and afterload conditions that apply to the left ventricle from the *in silico* model, and the desired patient's specific left ventricular mechanical properties in systole and diastole. The resulting target pressure and volume profiles for the left ventricle are calculated and applied to the phantom through an *ad hoc* controller and a hydraulic activation system made of DC motors and gear pumps. In turn, the pressure and flow signals measured in the *in vitro* left ventricle are sent back to the *in silico* cardiovascular model to update the overall hemodynamics at each iteration step and close the cardiovascular loop.

**FIG. 3.**
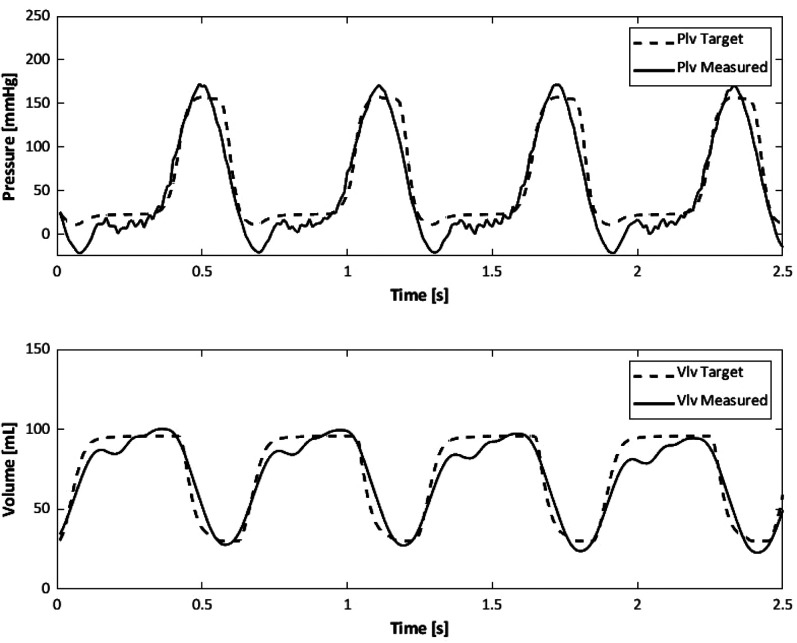
Example of the pressure (Plv) and volume (Vlv) profiles obtained for the HFpEF profile: data measured in the in vitro model (continuous line) and target trends (dashed line).

In Fig. 3 the target left ventricular pressure (Plv) and volume (Vlv) profiles are compared to those measured in the soft robotic left ventricle *in vitro*. Measured data are in accordance with the target trends although the end-systolic pressure shows a deeper negative peak due to the highly sensitive control system of the DC motors. Nonetheless, these results demonstrate the realism achieved in the simulations.

**FIG. 4.**
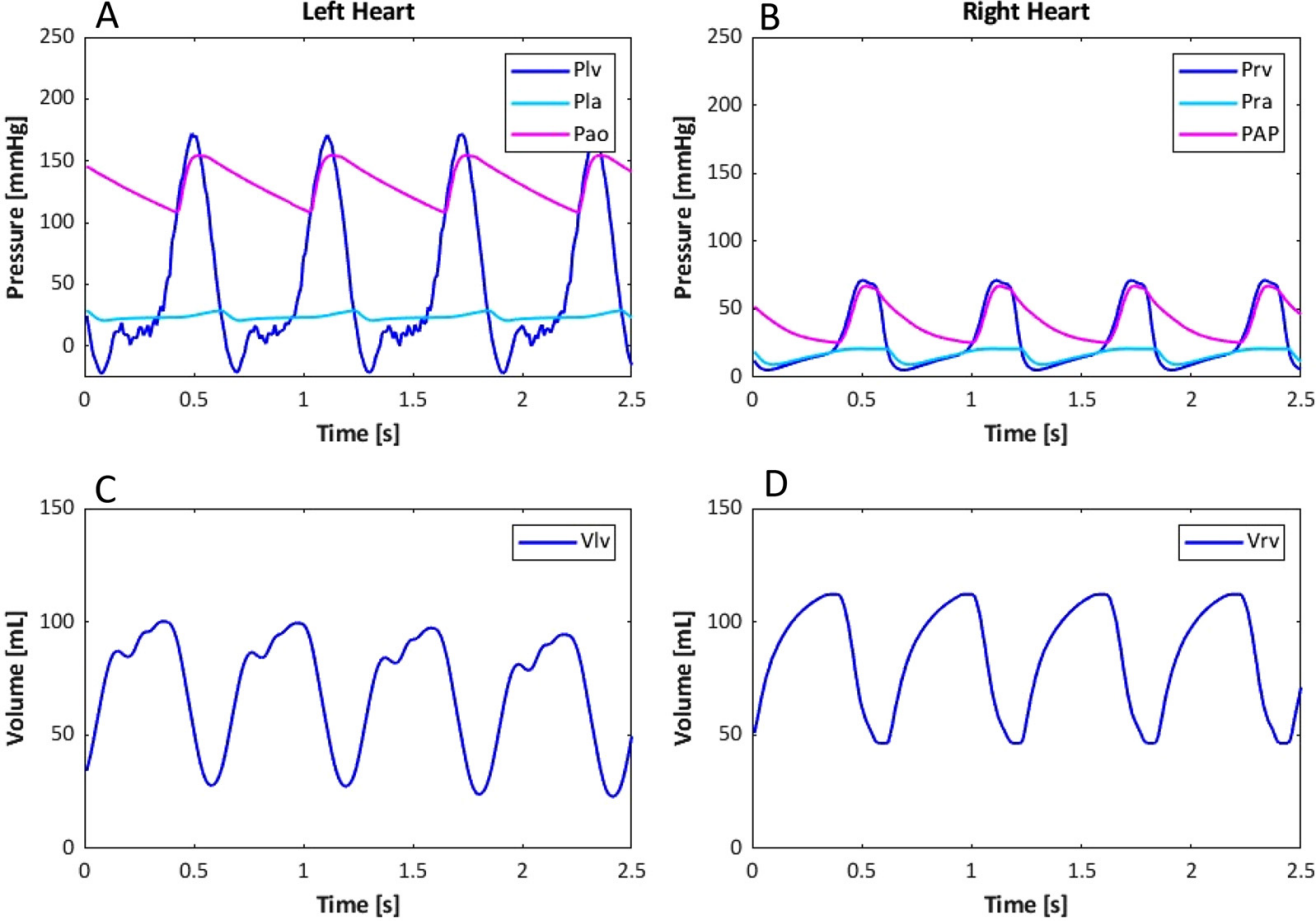
Simulated hemodynamics for the heart failure with preserved ejection fraction patient profile. (a) Left ventricular pressure (Plv) measured in the *in vitro* system; aortic pressure (Pao); and left atrial pressure (Pla) simulated in silico. (b) Right ventricular pressure (Prv), right atrial pressure (Pra), and pulmonary arterial pressure (PAP) simulated *in silico*. (c) Left ventricular volume (Vlv) measured in the in vitro system. (d) Right ventricular volume (Vrv) simulated in the in silico system.

In Fig. 4, the simulated hemodynamics of the left and right hearts of the HFpEF profile are shown as an example of the outcome of the personalization process of the hybrid simulator. All pressures and volumes are obtained from the *in silico* model, while the Plv and Vlv are measured in the *in vitro* left ventricle.

A comprehensive overview of the results obtained for the three patient profiles is reported in Table I. The comparison between the clinical and simulated data is included in terms of systolic/diastolic and average values, and in terms of nominal percentage errors. For all patient data, the errors are below the considered thresholds of 10% and 20% for the pressure and volumetric measurements, respectively.^29,30^ The error is higher for the systemic arterial pressure, as in the simulator this pressure is simulated in a single compartment that models the systemic circulation, while in the clinics it refers to the radial arterial one.^31^ Nevertheless, these results affirm the accuracy of the outputs obtained with the simulator and the versatility of the setup.

**TABLE I.** Clinical vs simulated patient data for the three patients' profiles. For the simulated data, the left ventricular pressure and volume are measured in the in vitro system, whereas the rest is simulated in the in silico system.

		HFpEF			Healthy			DCM		
		Clinical	Simulated	Error (%)	Clinical	Simulated	Error (%)	Clinical	Simulated	Error (%)
Cardiac output	(l/min)	4.1	4.4	7.3	4.9	4.8	2.0	4.3	4.5	4.7
Systemic arterial pressure systolic/diastolic(mean)	[mmHg]	172/69(102)	154/108(132)	10.5/56.5(29.4)	152/67(94)	137/87(113)	9.9/29.9(20.2)	145/71(96)	126/82(104)	13.1/15.5(8.3)
Pulmonary arterial pressure systolic/diastolic(mean)	[mmHg]	67/25(39)	67/25(41)	0/0(5.1)	32/14(20)	33/11(20)	3.1/15.4(0)	26/8(17)	29/11(18)	11.5/37.5(5.9)
Pulmonary capillary wedge pressure	[mmHg]	23	23	0	/	10	/	10	11	10
Right atrial pressure	[mmHg]	15	16	6.7	11	11	0	7	8	14.3
Left ventricular pressure systolic/diastolic	[mmHg]	/	157/11	/	/	140/82	/	/	130/4	/
Right ventricular pressure systolic/diastolic	[mmHg]	67/18	66/17	1.5/5.6	32/13	34/12	8.1/7.7	26/10	30/8	15.4/20
Left ventricular volumes systolic/diastolic	(ml)	32/93	28/94	12.5/0.5	83/145	83/144	0/0.9	113/184	115/189	1.8/2.9
Right ventricular volumes systolic/diastolic	(ml)	52/113	46/112	11.5/1.3	81/143	80/141	1.2/1.6	53/124	50/124	5.7/0.2

### Echocardiographic data

**FIG 5.**
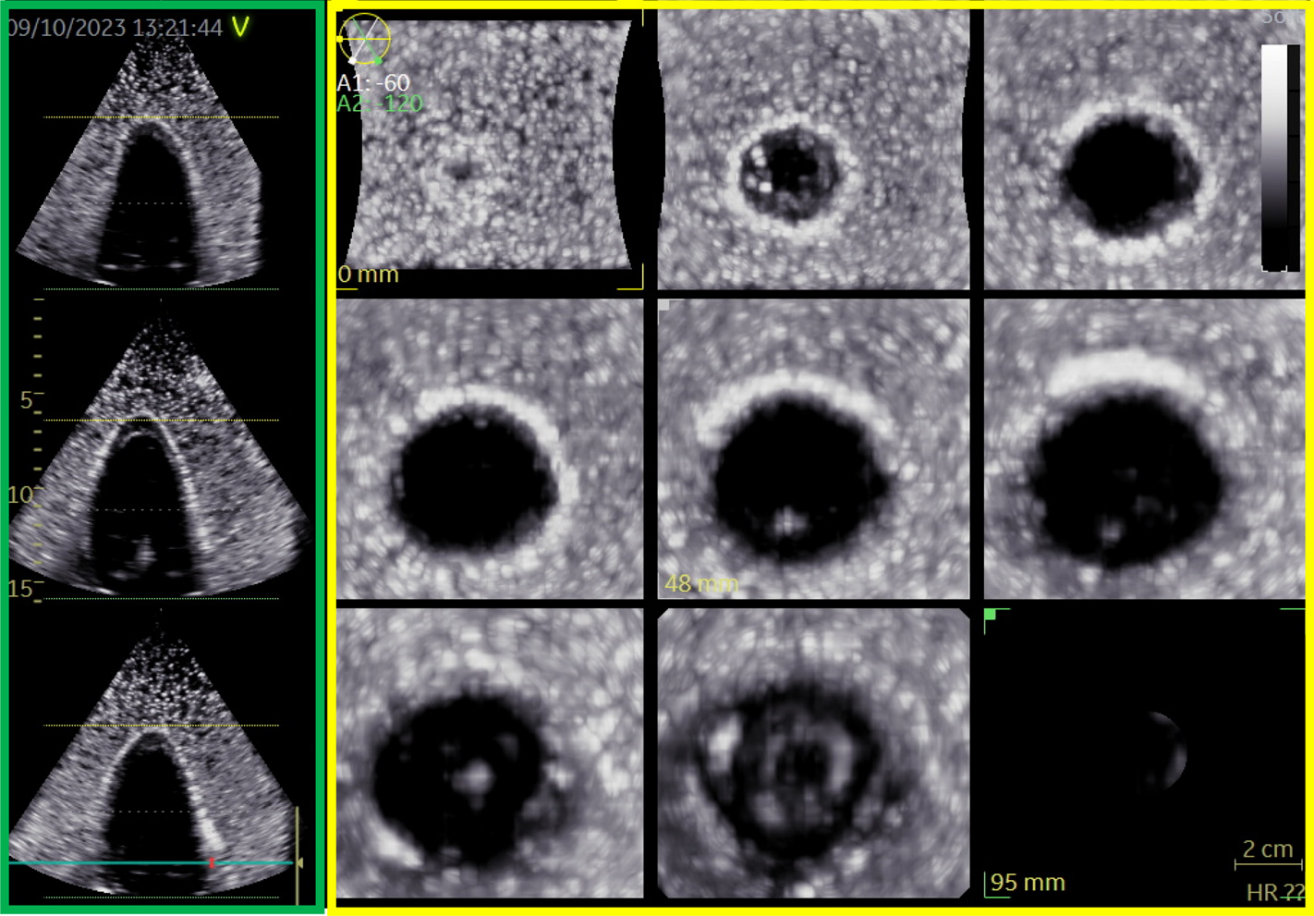
Echocardiographic 3D data of the soft robotic 3D left ventricular phantom of the HFpEF patient profile. In the green panel, the longitudinal axis of the phantom is shown. Each view is rotated by 60° counterclockwise with respect to the previous. These views are conventionally known as apical 4, apical 2. and apical 3 chamber view. In the yellow panel, the short axis views show the different levels of transverse planes of the phantom, starting from the apex, and going to the base, from the left upper panel to the right lower.

**FIG. 6.**
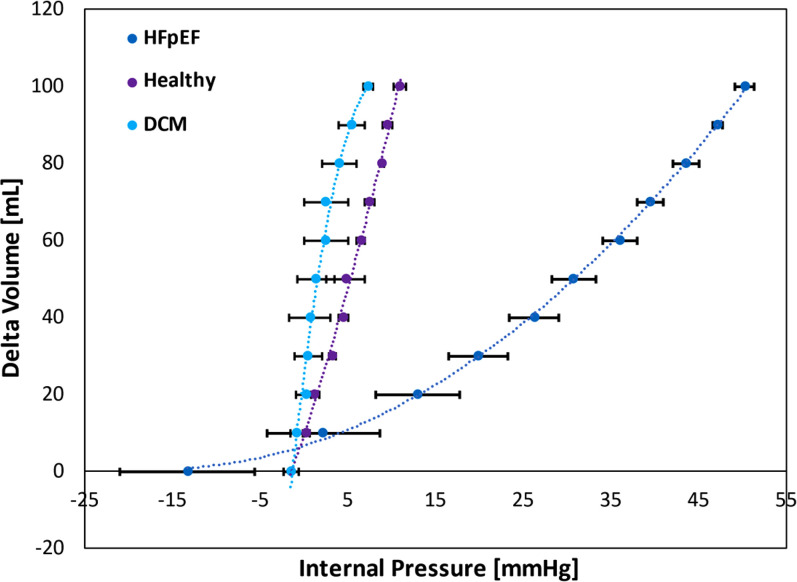
Compliance curves of the ventricular phantoms of the dilated cardiomyopathy (DCM), heart failure with preserved ejection fraction (HFpEF), and healthy profiles. Data points were acquired experimentally by increasing the volume inside the phantom and maintain the external pressure at 0 mm Hg. Data are shown as mean and standard deviation. With the dotted line, the second-order regression line is shown.

The 4D echocardiographic images of the left ventricular phantom were recorded from an apical view and stored for each patient profile. The images recorded in the HFpFE left ventricular phantom are shown in Fig. 5 as an example. Moreover, a video of the 4D echocardiography is added in the supplementary material.

Echocardiographic data were analyzed with the EchoPAC software (Version 204 GE Healthcare, Oslo, Norway) to retrieve the systolic and diastolic volumetric values for each left ventricular phantom. In Table II the comparison between echocardiographic and clinical volumetric values is shown. Echocardiographic data are in accordance with those measured in the clinical practice, with a maximal error of 12 ml for the systolic value of the HFpEF profile.

**TABLE II.** Comparison between the echocardiographic (Echo) data of the soft robotic left ventricle and the clinical volumetric data for the different patient profiles.

		HFpEF			Healthy			DCM		
		Clinical	Echo	Error (%)	Clinical	Echo	Error (%)	Clinical	Echo	Error (%)
Left ventricular end diastolic volume	[ml]	93	90	3.2	145	146	0.7	184	181	1.6
Left ventricular end systolic volume	[ml]	32	44	37.5	83	88	6.0	113	117	3.5

## DISCUSSION

With the advancement of sophistication of medical device technology, and the growth of people affected by cardiovascular diseases, a virtual explosion in the number of cardiovascular simulators to enable device testing has been registered, each with benefits and limitations contingent on the system's application.^5–7,11–27^

With the goal of testing medical devices in a high-fidelity physiological and anatomical condition, we developed a new test bench that combines different classes of simulators, to ultimately overcome their individual limitations. The hybrid simulator presented in this study combines the high flexibility of *in silico* systems, to the hydraulic interfaces of *in vitro* systems, where medical devices can be directly connected. The soft robotic left ventricle is implemented and controlled to represent patient-specific data in a high-fidelity anatomical model. The integration of a controllable soft robotic left ventricle into a cardiovascular simulator is a critical step forward toward the development of clinically relevant hemodynamic and biomechanical models personalizable to patient-specific conditions^27^ ranging from stiff left ventricles with a small cavity and a restrictive pattern of diastolic function, as in the HFpEF patient, to large ventricles with a predominately systolic dysfunction, as in dilated cardiomyopathy.

Unlike other models, our simulator enables an easy tunability of cardiovascular parameters toward the personalization of the system.^26,27^ Indeed, most of the cardiovascular system (except the left ventricle) is simulated numerically, thereby limiting the number of physical components that would need to be modified and/or replaced for model personalization. As such, the process of personalization of the hybrid simulator is largely simplified and consists of the tuning of numerical parameters and the development of a compliant patient-specific left ventricular model through a mold-casting technique.

To demonstrate the flexible nature of the hybrid simulator, three different patient profiles, characterized by very different hemodynamics and left ventricular mechanical properties, were represented in this study (Fig. 9 in Appendix). The advanced technology implemented in the simulator not only gave the possibility of simulating physiological pressures and flow profiles but also of representing realistic volumes as shown in the video in the supplementary material. Indeed, the activation method implemented enables to regulate the dilation and contraction of the soft robotic left ventricle and to reproduce concomitant pressure and volume profiles over a cardiac cycle in realistic range of values (HRs from 61 to 79 bpm, Plv from 0 to 157 mm Hg, and Vlv from 44 to 181 ml). Overall, the results demonstrated high levels of accuracy, realism, and versatility, in line with the KPIs set for the system.

The soft robotic left ventricle is developed using PVA, an echogenic, inexpensive, and easy-to-use material characterized by interesting mechanical properties. The stiffness of the material can be easily adjusted by altering either the number of freezing and thawing cycles or the percentage of PVA in the solution. In this study, a clinically available imaging technique to define the volume change in the cardiac cycle was used. The echogenicity of the PVA phantoms enabled a good definition of the volume throughout the cardiac cycle, as well as an adequate imaging and delineation of both the internal and external border of their walls. Volumetric data were in accordance with the clinical data, with errors comparable to those found in other studies.^30^ As such, our cardiovascular simulator represents a good test bench to verify and validate imaging techniques, with a particular interest in 3D ultrasound imaging techniques.^32^

Eventually, the methodology described in this paper could be broadened to different anatomical cardiovascular sites, such as the atria and the right ventricle. The authors decided to start with the left ventricle, which seemed to be the most challenging cardiac site to represent given the complex mechanical properties, to pave the way for high-fidelity patient-specific tunable anatomical models to be used for a variety of purposes.

Despite the many advantages offered by the hybrid simulator, there are a few limitations to consider. Given the authors' choice to have a limited number of physical elements, no valves are included in the anatomical left ventricular phantom, whose actions are simulated by an alternating bidirectional flow into the phantom using the gear pump. This characteristic does not affect the representation of the left ventricular pressure and volumes over a cardiac cycle but does not create the physiologically distinct inflow and outflow patterns. However, the implementation of valves could be envisaged in the future to further broaden the application of the simulator to the testing of heart valve prostheses and to the investigation of flow patterns in left ventricular anatomies.

Improvements to this study would consider a better connection of the anatomical soft robotic element for a more physiologic representation of the contraction, with the valvular plane moving toward the apex.

Given the physiological and anatomical high fidelity of the hybrid simulator and the flexibility to represent a wide range of patient profiles, we envision diverse applications in the field of medical technology. This includes testing of a broad class of medical devices, such as implantable mechanical circulatory support systems like ventricular assist devices, or imaging techniques like 3D ultrasound imaging techniques. The hybrid simulator is versatile and can serve purposes such as optimizing medical device design and related embedded control algorithms and targeting the patient population that would benefit most. In clinical scenarios, the hybrid simulator could function as a decision support system for device selection and/or contribute to the characterization of personalized therapies.

## METHODS

The cardiovascular simulator is a hybrid system that combines both an *in vitro* and an *in silico* model, connected to each other in real-time under the LabVIEW environment (LabVIEW 2019 SP1, National Instruments, Austin, Texas). A/D and D/A converters are used to exchange data between the *in vitro* and the *in silico* models. The different components of the simulator are detailed in the next sections.

### *In silico* model

The *in silico* model is a lumped parameter representation of the closed-loop cardiovascular system, including atria, ventricles, and systemic and pulmonary circulations (Fig. 8 in Appendix). The ventricles are modeled with a time-varying elastance model following the Frank–Starling law.^33^ The pressure–volume relationships of both the left and right ventricles are defined by an exponential function during the diastolic phase expressed as

$$P_{V}\left(t\right)=a{\cdot e}^{b\cdot \;V_{V}\left(t\right)},$$ (1)

where *P_V_* and V_V_ are the ventricular pressure and volume, respectively, and *a* and *b* are constant values, different for the left and right ventricles.

The systolic ventricular pressure–volume relationship is modeled with a time varying elastance model^33^ implemented as follows:

$$P_{V}\left(t\right)=E_{V,s}\cdot v_{C}\left(t\right)\cdot \left(V_{V}\left(t\right)-V_{0}\right),$$ (2)

where *v_C_* is the ventricular contraction function ranging between 0 in diastole and 1 in systole, *E_V,s_* is the ventricular systolic elastance, and *V_0_* is the zero pressure filling volume, with *E_V,s_* and *V_0_* differing for the left and right ventricles.

Finally, a Windkessel model is used for both systemic and pulmonary circulations.^34^

### *In vitro* model

The *in vitro* model consists of a hydraulic chamber where the soft robotic left ventricle is implemented. The chamber is filled with water and is activated by 2 custom-made gear pumps (Sirris, Brussels, Belgium) each connected to a DC motor (Maxon Motor Ag, Sachseln, GE). The gear pumps were designed to achieve a maximum flow of 38 L/min and a displacement of 15 cc/rpm. Motors with a nominal speed higher than 2500 rpm were selected. These enable the simulation of the hemodynamic profile of a healthy left ventricle. One gear pump is used to control the pressure inside the left ventricular model (Pint), whereas the second gear pump is used to control the external pressure (Pext) as explained in the next sections. Pressure sensors are used to measure both Pint and Pext to have a closed feedback loop to control the motors (Fig. 8 in Appendix) (PPG Honeywell, Columbus, OH, USA for Pext and AP023 Autosen GmbH, Essen, Germany for Pint). The internal pump provides bidirectional flow in and out of the 3D left ventricular phantom, so no mitral and aortic valves are implemented. The mechanical properties of the aortic and mitral valves (in terms of opening/closing and resistance) are represented in the *in silico* model.

### Soft robotic left ventricle

The anatomy of the left ventricle was extracted from the CMR images. Images were acquired with a Philips Achieva 1.5-T CMR with a 5-element phased-array coil (Philips Medical Systems, Best, the Netherlands) and analyzed with a software program developed in-house (RightVol, Leuven, Belgium).^35^ Briefly, left ventricular endocardial contours were manually traced on the short-axis images, and the points of transection with the horizontal long-axis plane were indicated, thus enabling constant referencing of the atrioventricular valve plane. Trabeculations and papillary muscles were considered part of the ventricular blood pools, and volumes were calculated by a summation of disks.

The soft robotic left ventricle is created using the mold-casting technique as shown in Fig. 2.^22^ As such, the 3D geometry of the left ventricular systolic phase is extracted from a CMR image, segmented and imported in the Blender 4.0 modeling software (Blender Foundation, Amsterdam, The Netherlands) to design both the internal and external molds.

**FIG. 7.**
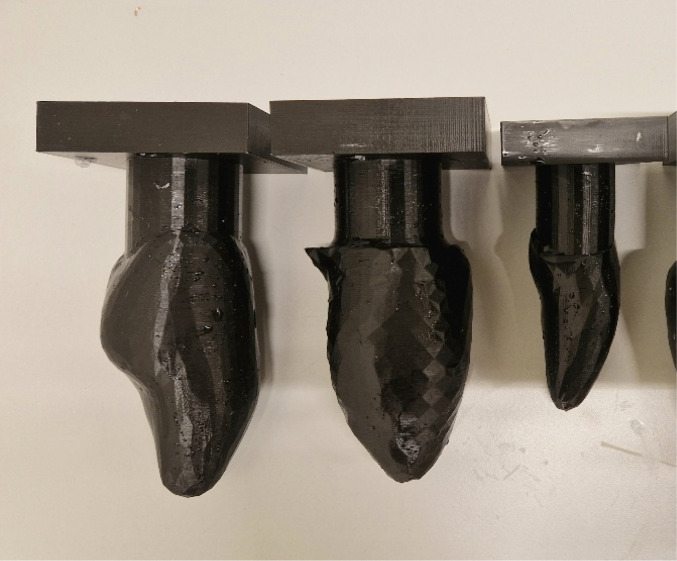
Internal molds of the three soft robotic left ventricular models.

The internal mold represents the left ventricular cavity during the systolic phase with only one access point functioning both as inflow and outflow for the gear pump (Fig. 7 in Appendix). The external mold is defined as the negative version of the dilated internal cavity to guarantee a gap between the two molds equal to the desired thickness. In this study, a constant thickness of 10 mm is considered. An ellipsoid of 10 mm is added at the top of the negative dilated version of the internal cavity for the connection to the *in vitro* system. Finally, an access point was designed in the external mold from which to pour the solution of PVA.

#### Material of the soft robotic left ventricle

A water solution of glycerol (10% w/w) and PVA(10% w/w) is chosen as the material. For the creation of such a solution, the powder of PVA (Kuraray Europe GmbH, Hattersheim am Maim, Germany) is initially mixed with a solution of water and glycerol at 80 °C until a transparent solution is obtained. The solution is then poured in the mold and subjected to 2 freeze–thaw cycles. One cycle consists of 24 h in the freezer at −20 °C and 24 h of thawing at environment temperature. The number of cycles was determined to achieve mechanical and acoustic properties close to those of a left ventricle.^36^

Once the left ventricular model is obtained, no particular attention had to be given to the removal process of the internal mold from the PVA model, given the shape of the ventricles. A static characterization is then performed to measure the compliance of the left ventricular phantom. Starting from the zero pressure filling volume (V_0_), the phantom is filled in steps of 10 ml of water, until reaching 100 ml, by using a syringe connected to the model's inflow/outflow tract. For each volume step, the Pint is measured. Tests are repeated for different values of the Pext ranging between -50 mm Hg and 150 mm Hg, with a step of 25 mm Hg. Finally, the compliance of the material (C) is derived from the regression line of the pressure–volume (PV) measurements obtained with the static characterization.

#### Activation method of the soft robotic left ventricle

According to physiology, the compliance of a left ventricle changes over a cardiac cycle, as the ventricle shows both an active behavior during the systolic contraction and a passive behavior during the diastolic filling.

To recreate a realistic PV loop of the left ventricle with a passive material, the soft robotic left ventricle is activated using 2 gear pumps. One gear pump is connected to the inflow/outflow tract of the 3D phantom, and it is used to recreate the left ventricular volume. The other gear pump pushes liquid in/out to control the pressure in the hydraulic chamber (Pext) where the left ventricle is embedded. According to the compliance value measured in the soft robotic left ventricle and given the desired left ventricular volume and pressure during the cardiac cycle, an algorithm was developed to derive the Pext necessary to recreate the match between Plv and Vlv in the 3D model.

Pext is obtained as follows:

$$P_{\text{ext}}=\mathrm{Pint}-\frac{V_{t}-V_{0}}{C}=\mathrm{Plv}-\frac{V_{lv}-V_{lv,s}}{C},$$ (3)

where V_t_ is the target volume, equal to the left ventricular volume during the simulation, and V_0_ is the initial volume of the soft robotic left ventricle, equal to end systolic volume of the patient V_lv,s_. This activation method guarantees the application of an even force on the surface of the soft robotic left ventricle and can be adapted to a wide range of (patho-)physiologies.

### Verification of the model

#### Clinical data

The clinical data used in this study were retrospectively collected from a dataset of patients enrolled in the study approved by the Ethics Committee of UZ Leuven (B322201214035).^37^ A total of three patients were selected to prove the ability of the novel hybrid simulator to represent different left ventricular models. Namely, a patient with DCM, one with HFpEF, and a healthy volunteer. The patients are characterized by different mechanical properties of the left ventricle. The DCM is characterized by a poor contractility and an impaired relaxation. The HFpEF is characterized by preserved contractility but with stiff diastolic function. Finally, the healthy heart is characterized by a normal contractility and diastolic function. Each patient underwent a CMR scan with a concomitant pulmonary and radial artery catheterization and an electrocardiogram (ECG). As such, for each patient, the volume of the left and right chambers is known, as well as the radially measured arterial pressure, pulmonary arterial pressure (PAP), right atrial pressure (RAP), pulmonary capillary wedge pressure (Pwedge), the HR, and the cardiac output (CO).

#### Model personalization

**FIG. 8.**
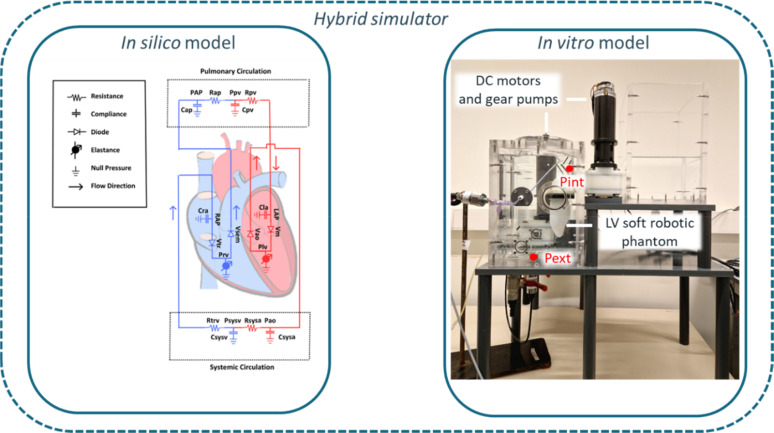
Hybrid simulator. The hybrid simulator combines an in silico and an in vitro system. On the left, the electrical equivalent circuit of the cardiovascular system implemented in the in silico model. The left and right atria are represented as linear compliances (Cla and Cra), left and right ventricles are represented as a nonlinear compliance during diastole [Eq (1)] and a time-varying elastance model during systole [Eq. (2)]. Heart valves are represented by a diode (Vm, Vao, Vsem, and Vtr). The pulmonary arterial circulation is represented by a compliance (Cap) and a resistance (Rap). Pulmonary venous circulation is represented with a compliance (Cpv) and a resistance (Rpv). The systemic arterial circulation is represented by a compliance (Csysa) and a resistance (Rsysa). Systemic venous circulation is represented with a compliance (Csysv) and a resistance (Rsysv). RAP: right atrial pressure; Prv: right ventricular pressure; PAP: pulmonary arterial pressure; Ppv: pulmonary venous pressure; LAP: left atrial pressure; Plv: left ventricular pressure; Pao: aortic pressure; Psysv: systemic venous pressure. On the right, the in vitro model. The left ventricular (LV) soft robotic phantom is implemented in the hydraulic chamber. Two gear pumps and DC motors are connected to the hydraulic chambers. The in vitro system has 2 pressure sensors (red dots). One sensor measures the internal pressure (Pint) of the LV phantom, whereas the other measures the external pressure (Pext). (Appendix).

**FIG. 9.**
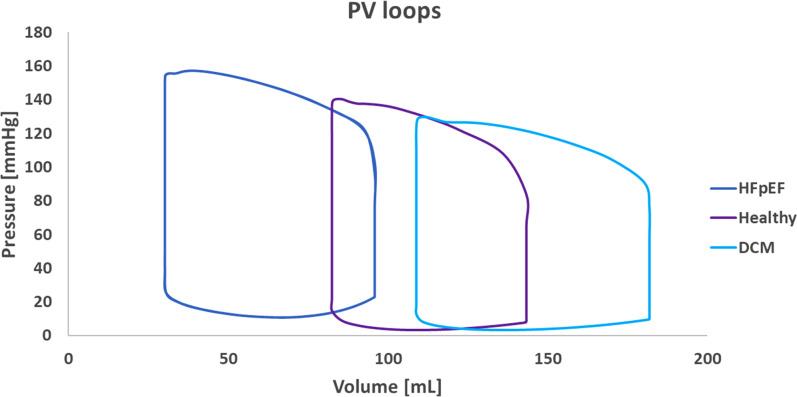
PV loops of the dilated cardiomyopathy (DCM), heart failure with preserved ejection fraction (HFpEF), and healthy profile simulated in the study (Appendix).

The three left ventricular phantoms are developed starting from the CMR images of each patient following the aforementioned procedure. For each patient, the correspondent hemodynamic profile is characterized starting from the clinical measurements according to the protocol of personalization of the cardiovascular *in silico* model described in Fresiello *et al.*^38^ Briefly, the end-systolic and end-diastolic pressure volume relationships for both the left and right ventricle are measured starting from the pressure measurements of the arterial lines and the volumes extracted from the CMR data. The HR of the patient is retrieved from the ECG data. The pulmonary vascular resistance (PVR) and the systemic vascular resistance (SVR), which are measured based on Ohm's law and comply with clinical standards, are expressed as

$$SVR=\frac{\mathrm{MAP}-\mathrm{RAP}}{CO},$$ (4)

$$PVR=\frac{\mathrm{PAPm}-\mathrm{Pwedge}}{CO},$$ (5)

where MAP is the mean systemic arterial pressure and PAPm is the mean pulmonary arterial pressure. All the other parameters shown in Fig. 8, that were not measured in the patients, were taken from literature.^39–41^ In Table III the parameters measured starting from clinical data are shown.

#### Experiments

For each patient profile, the simulator was activated. Measurements of both the *in silico* and *in vitro* data were recorded simultaneously over 20 cardiac cycles. Namely, the Pint, Pext, and the pumps' flows were recorded from the *in vitro* model. From the *in silico* model, the pressure, volumes, and flows of the cardiovascular system were recorded.

Moreover, a GE ultrasound probe, suitable for echocardiography (4Vc) was connected to the simulator to acquire 4D volumetric data of the soft robotic left ventricle from a longitudinal (apical) view. GE Vivid E95 (GE Healthcare, Oslo, Norway) was used, and the analysis was performed with EchoPAC version 204. In particular, for each patient profile, the contours of the soft robotic left ventricle were manually defined by an experienced echocardiographer both at end systole and end diastole, defined as the frame with the visually assessed smallest and largest internal volume of the phantom, respectively.

#### Data analysis

For each patient profile, data from the hybrid simulator were compared to the clinical data. Hemodynamic and volumetric data were recorded and averaged over 20 cardiac cycles. The volumes of the 3D left ventricular phantom were extracted from the integration of the flow of the internal and external pump obtained from a postprocessing of the data using MATLAB. The average maximal value and minimal value of the volume were then used to represent, respectively, the end-diastolic and end-systolic left ventricular volume.

The performance of the hybrid cardiovascular simulator with a soft robotic left ventricle was assessed using the following key performance indicators (KPIs):

• Accuracy
• Realism
• Versatility

Accuracy was evaluated by comparing hemodynamic and echocardiographic data measured in the hybrid simulator to clinical data. The error is calculated as a nominal error percentage. The authors considered 10% and 20% as thresholds for good agreement between simulations and clinical data, for hemodynamic and echocardiographic data, respectively. These thresholds were decided considering the error usually affecting these clinical measurements.^29,30^

The realism of the simulation was defined as the ability of the simulator to replicate both the systolic and diastolic function of the left ventricle, as well as in creating high-fidelity pressure and volume waveforms.

Finally, the versatility of the simulator was assessed by the ability of the simulator to recreate different hemodynamic scenarios and different anatomical models.

## SUPPLEMENTARY MATERIAL

See the supplementary material for details the video 1: 3D echocardiography of the soft robotic left ventricle for a cardiac cycle. The video shows the 3 imaging planes and a 3D reconstruction of the ventricular shape. Finally, the waveform of the reconstructed ventricular volume is shown.

## Data Availability

The data that support the findings of this study are available from the corresponding author upon reasonable request.

## APPENDIX: DATA AND VISUAL SUPPORT

Compliance curves of the ventricular phantoms of the dilated cardiomyopathy (DCM), heart failure with preserved ejection fraction (HFpEF), and healthy profiles (Fig. 6). Internal molds of the three soft robotic left ventricular models (Fig. 7). Hybrid simulator combining an *in silico* and an *in vitro* system (Fig. 8). PV loops of the dilated cardiomyopathy (DCM), heart failure with preserved ejection fraction (HFpEF), and healthy profile simulated in the study (Fig. 9)[App app1]. Input parameters of the in silico model for the 3 patient profiles taken from clinical data (Table III). [App app1]

**TABLE III.** Input parameters of the in silico model for the 3 patient profiles taken from clinical data. (Appendix).

		HFpEF	Healthy	DCM
Heart rate	(bpm)	67	79	61
Pulmonary vascular resistance	(WU)	5.2	2	1.6
Systemic vascular resistance	(WU)	26.6	20	20.5
Left ventricular end-systolic elastance	(mmHg/ml)	5.4	1.7	1.2
Left ventricular diastolic function's parameters [Eq. (1)]	*a_L_* (mmHg)	0.24	0.07	0.05
	*b_L_*	0.04	0.033	0.029
Right ventricular end-systolic elastance	(mmHg/ml)	1.25	0.44	0.6
Right ventricular diastolic function's parameters [Eq. (1)]	*a_R_* (mmHg)	0.24	0.07	0.07
	*b_R_*	0.03	0.034	0.038
